# Being Part of an Editorial Board: Implications and Scope for Scientific Communication and Personal Academic Development*

**DOI:** 10.17533/udea.iee.v41n3e13

**Published:** 2023-10-31

**Authors:** R. Mauricio Barría P.

**Affiliations:** 1 RN, M.Sc, Ph.D. Director of the Institute of Nursing, Faculty of Medicine, Universidad Austral de Chile. email: rbarria@uach.cl Universidad Austral de Chile Faculty of Medicine Universidad Austral de Chile Chile rbarria@uach.cl

**Keywords:** periodicals as topic, editorial policies, scholarly communication, scientific and technical publications, publicaciones periódicas como asunto, políticas editoriales, comunicación académica, publicaciones científicas y técnicas, publicações periódicas como assunto, políticas editoriais, comunicação acadêmica, publicações científicas e técnicas

## Abstract

**Objective.:**

From my experience as a member of the editorial board of the journal *Investigación y Educación en Enfermería*, the implications and scope of participating in this entity and the mutual and reciprocal benefits of this academic interaction between members of the editorial board and the journal are explained.

**Content synthesis.:**

The key elements on operation, integration, tasks, and responsibilities of editorial boards to disseminate scientific research in different disciplines are analyzed and described, highlighting the rigor and commitment to academic ethics that allows guaranteeing the credibility of the contents published and topics addressed by a journal within a context of high competitiveness and risk of breaches of academic and scientific probity and ethics.

**Conclusion.:**

Integrating an editorial board requires developing a fundamental role that implies a series of commitments and challenges that must be addressed with professionalism and ethics to guarantee the quality and prestige of the academic publication. In this task, achievements and goals are reached for the journal, as well as academic benefits for the editorial board members.

## Resumen

**Objetivo.:**

A partir de mi experiencia como integrante del comité editorial de la revista Investigación y Educación en Enfermería, se exponen las implicancias y el alcance de participar en esta entidad y los beneficios mutuos y recíprocos de esta interacción académica entre los miembros del comité editorial y la revista.

**Síntesis del contenido.:**

Se analizan y describen los elementos claves sobre funcionamiento, integración, tareas y responsabilidades de los comités editoriales para la difusión de investigaciones científicas en las distintas disciplinas, resaltando el rigor y compromiso con la ética académica que permite garantizar la credibilidad de los contenidos publicados y las temáticas abordadas por una revista en un contexto de alta competitividad y de riesgo de faltas a la probidad y ética académica y científica.

**Conclusión.:**

Integrar un comité editorial exige desarrollar un papel fundamental que implica una serie de compromisos y desafíos que deben abordarse con profesionalismo y ética para garantizar la calidad y el prestigio de la publicación académica. En esta tarea se alcanzan logros y metas tanto para la revista, así como se obtienen beneficios académicos para los integrantes del comité editorial.

## Resumo

**Objetivo.:**

Com base na minha experiência como membro do conselho editorial da *Revista Investigación y Educación en Enfermería*, são apresentados as implicações e o alcance da participação nesta entidade e os benefícios mútuos e recíprocos desta interação acadêmica entre os membros do conselho editorial e a revista.

**Síntese de conteúdo.:**

São analisados ​​e descritos os elementos-chave sobre o funcionamento, integração, tarefas e responsabilidades dos comités editoriais para a divulgação da investigação científica nas diferentes disciplinas, destacando o rigor e o compromisso com a ética académica que permite garantir a credibilidade dos conteúdos publicados e os temas abordados por uma revista em um contexto de alta competitividade e de risco de violação da probidade e da ética acadêmica e científica.

**Conclusão.:**

Integrar um comitê editorial exige desenvolver um papel fundamental que envolve uma série de compromissos e desafios que devem ser enfrentados com profissionalismo e ética para garantir a qualidade e o prestígio da publicação acadêmica. Nessa tarefa são alcançadas conquistas e metas para a revista, bem como benefícios acadêmicos para os membros do comitê editorial.

## Introduction

Editorial boards of academic journals play a fundamental role in scientific development and advancement, as well as in the growth of disciplines and professions by providing scientific support that backs their actions. These boards, comprised by experts in specific fields, are responsible for reviewing and evaluating manuscripts submitted for possible publication based on the policies and regulations defined according to their action setting and scope. Given the aforementioned, the quality and visibility of the contents of a journal are closely linked to the editorial process, and it is, therefore, necessary for editorial policies to define criteria for accepting manuscripts, technical conditions, regulatory standards, peer-review model, frequency of publication, among other aspects.^1^

The editorial board is generally made up by outstanding members in the journal’s particular field. These should be research peers whose criteria are highly respected within the journal’s discipline; otherwise, their decisions might not be considered valid. Board members act as journal ambassadors and much of their quality is judged by the merits and academic credentials of its staff.^2^ This way, based on the academic support of each member, one of the most important objectives of the editorial board members is to improve the impact of their journals and, in addition, thanks to this academic prestige, they seek to improve the quality of their journals.^3^^,^^4^ Consequently, the importance of the scientific merit to select the editorial board members is highlighted as fundamental criterion for a sound editorial evaluation of the manuscripts the journal receives and publishes.^5^

As reported by the Springer Nature website (international scientific publisher in the field of science and medicine),^2^ the functions of the editorial board in its advice and support to the editor include: advising on the direction of the journal by giving feedback on published issues and suggesting potential topics and authors; providing content by writing occasional editorials and other short articles; contacting and suggesting possible contributors; conducting peer review, helping to identify other peer reviewers, and providing second opinions about articles in case of conflict among reviewers.

Currently and for some years now, aware of the high competition to attract research work and articles of interest for a journal, a certain laxity or flexibility has been detected in the evaluation criteria to sustain the viability of the journal. Given this panorama, it is when a greater need exists for high-quality editorial boards with sufficient demanding standards, so that contents published have support that guarantees the veracity of what is published and is useful for the specific science community and society. This article, from my experience as Editorial Board member of the journal *Investigación y Educación en Enfermería*, seeks to expose the implications and scope of participating in this entity and the mutual and reciprocal benefits of this academic interaction. 

## The transition to becoming an editorial board member

In the life of an academic, different stages are undertaken and different functions are developed. It is likely that, in many cases, the attraction to becoming a university professor, in addition to teaching, lies in the idea of conducting research and generating knowledge in specific disciplinary settings. The development of skills for research and scientific production based on works presented at conferences, as well as the preparation of manuscripts for publication, constitute a basic point to meet an academic standard sufficient to be considered in any journal scientific or editorial board. 

**Figure 1:**
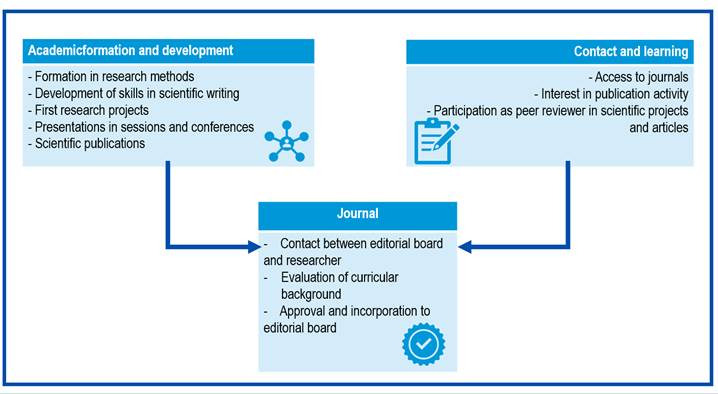
Necessary elements and conditions to integrate an editorial board.

Further, in the construction of a specific academic profile, one comes across attractive journals and on which one projects the intention of being part of them. In my experience, in the early 2000s, I had the opportunity to hold an issue of the journal *Investigación y Educación en Enfermería*. Due to the themes and quality of the works, as well as the editorial line and aspects of style, my aspiration arose to be part of the Journal. In this experience and from these two perspectives, two approaches come together as a basis for meeting the basic conditions to be part of an editorial board. As illustrated in Figure 1, essential conditions and experiences are needed to meet the requirements to join these boards.

## Learning and assessment of the rigor of a scientific journal

From the experience in *Investigación y Educación en Enfermería*, and on a retrospective analysis of the evolution during 15 years (since my incorporation in 2008) to what it is today, the actions and strategies the Journal has undertaken to achieve the position of excellence it boasts as a high-impact publication, and its incorporation into reference bases of the highest prestige, become evident.

What lies behind these achievements exposes all the integrated work involving actions from the Institution, policies defined by directors and editors at different times, and the joint work with the editorial board and the team of peer reviewers. This results in a highly rigorous operation with a solid and organic structure to achieve higher standards of management and efficiency. A crucial point has been the rigorous selection of its editorial and scientific team, incorporating the most-suitable people.

We can, thus, recognize that journals aspiring to the highest standards need to form an editorial board in which at least these conditions are met:

- Academic reputation. Board members must be recognized in their respective fields through their experience and contributions to research. Academic reputation contributes to the journal’s credibility and attracts quality authors and researchers.
- Diversity of experience and knowledge of its members. A solid editorial board must have members who represent a wide range of experiences and perspectives. Diversity in terms of fields of study, academic background, and methodological approaches enrich editorial decision making and guarantee the journal’s relevance for a broad audience.
- Ethics and professionalism. It is fundamental for the editorial board members to be committed with rigorous ethical standards and be able to transparently address possible conflicts of interests.
- Commitment and availability. Being part of an editorial board implies a significant investment of time and effort. Members must be willing to review manuscripts, participate in editorial discussions, and provide guidance to authors and reviewers. Long-term commitment is essential for the journal’s success.
- Review skills. Board members must have solid review skills to evaluate critically manuscripts submitted to evaluation. They must be capable of identifying methodological weaknesses, bias, errors, and limitations of works received.

**Figure 2:**
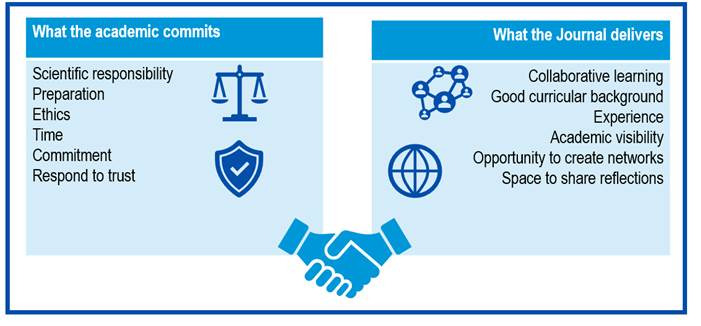
Reciprocal relation between the academic and the journal in the editorial board.

With the foregoing, and from an analysis of a reciprocal relation, it is possible to establish that in the interaction between editorial board members and the journal commitments and mutual benefits are integrated (Figure 2).

An interesting view is one that exposes the benefit of integrating an editorial board for the institutions in which the board member works, as well as for the institutions from which they graduated, both in terms of visibility and as an indicator of other forms of productivity. Editorial board representation provides a measure of the influence and visibility of any institution in a given discipline. In this, competing views highlight the influence, visibility and productivity of research for the institutions employing the editorial members, while others have argued that the alma mater of an editorial board member is a more stable indicator because it does not change as does occur with the labor institution.^6^

## Essential tasks and commitments of the editorial board

In the combination of the elements proposed, some key reasons emerge that demonstrate the importance of an integrated operation that combines the journal’s interests, as well as the interests and aspirations of the editorial board members. For this to occur, it is understood that the following operating conditions are met:

- Guarantee of quality and scientific rigor: editorial boards are responsible for evaluating the scientific quality of manuscripts. This includes verifying the methodology, results, and interpretation of the findings.
- Impartial evaluation: editorial boards must ensure that the peer review process is fair and impartial. This involves selecting appropriate reviewers and ensuring that judgments are based on academic and scientific merit, rather than personal prejudice or bias.
- Academic integrity: editorial boards play a crucial role in detecting and preventing plagiarism, duplication of publications, and other forms of scientific misconduct. Their surveillance contributes to maintaining the integrity of scientific literature.
- Support to the scientific community: upon providing constructive feedback to authors, editorial boards help researchers improve their work and contribute more meaningfully to the field. They also promote effective scientific communication.

In summary, editorial boards and their members themselves play an essential role in the scientific publication process by ensuring the quality, integrity, and relevance of articles published. Their experience and commitment with scientific excellence are fundamental for the advancement of research and knowledge in various academic disciplines. A relevant point within the current context is precisely the existence of journals that, due to different reasons and objectives, do not meet the basic standards to regulate a publication of poor or clearly invalid content. This occurs, for example, with journals that do not have sufficient technical resources, to which low-quality manuscripts are submitted, that have no international recognition, and have a limited number of editors or reviewers. In these cases, less rigorous review processes are usually adopted to complete issues of the journal in its struggle for survival.^6^ The other threat is constituted by predatory journals, which are journals that only seek a profitable business at the expense of researchers attracted by the idea of publishing quickly and massively in exchange for a publication fee, but with minimal control and regulation of what is published.^7^ Predatory journals already exist widely in nursing and bring along lack of transparency regarding editorial processes. Reviewers and editors of these journals contribute to the problem by lending their names to a dubious journal, sometimes ignoring that their names are being used. Due to the aforementioned, although being invited to participate as a reviewer, editorial board member or even editor can be flattering, one must make sure that it is a publication that deserves affiliation and that adds and does not take away from the curriculum.^8^

## Conclusion

This article sought to capture the implications and scope of what it entails to be part of an editorial board and to give an account of the functions and tasks that, as a team, it must undertake and develop to provide guarantees of scientific credibility and the contribution that journals can and should provide to a particular academic community and to society at some level. Participating in an editorial board of a scientific journal is a significant responsibility that entails various implications for the board members and for the scientific community as a whole. This fundamental role implies a series of commitments and challenges that must be addressed with professionalism and ethics to guarantee the academic publication’s quality and prestige. But it should be recognized that this task not only reaches achievements and goals for the journal, but also provides the opportunity to obtain academic benefits for the editorial board members.

Note: this article is based on the presentation: Fifteen Years in the Editorial Board: Challenges and Achievements for Joint Growth presented in the activity commemorating the journal *“Investigación y Educación en Enfermería 40 years disseminating knowledge*”, August 2023; Medellín (Colombia).

## References

[1] 1. Fernández Bajon MT, Guerra González JT. Transparencia editorial en revistas científicas mexicanas de educación: hacia una gestión integral de las políticas editoriales en las publicaciones periódicas científicas. Investig. Bibl. 2021; 35(87):13-32.

[2] 2. Springer Nature. Editorial Boards [Internet]; 2023. Available from: https://www.springer.com/gp/authors-editors/editors/editorial-boards/

[3] 3. Wu D, Lu X, Li J, Li J. Does the institutional diversity of editorial boards increase journal quality? The case economics field. Scientometrics. 2020; 124:1579-97.

[4] 4. Wu D, Li J, Lu X, Li J. Journal editorship index for assessing the scholarly impact of academic institutions: An empirical analysis in the field of economics. J. Informetr. 2018; 12(2):448-60.

[5] 5. Tutuncu L. Editorial board publication strategy and acceptance rates in Turkish national journals. J. Data Inf. Sci. 2023; 8(4):1-35.

[6] 6. Musambira GW, Hastings SO. (2008) Editorial Board Membership as Scholarly Productivity: An Analysis of Selected ICA and NCA Journals 1997-2006. Rev. Commun. 2008; 8(4):356-73.

[7] 7. Barría RM. Scientific rigor, the ethics of publications and the temptation of predatory journals. Invest. Educ. Enferm. 2018; 36(3):e01. 10.17533/udea.iee.v36n3e01 31083847

[8] 8. Oermann MH, Conklin JL, Nicoll LH, Chinn PL, Ashton KS, Edie AH, Amarasekara S, Budinger SC. Study of Predatory Open Access Nursing Journals. J. Nurs. Scholarsh. 2016; 48(6):624-32. 10.1111/jnu.12248 27706886

